# Influence of the *CXCL1* rs4074 A Allele on Alcohol Induced Cirrhosis and HCC in Patients of European Descent

**DOI:** 10.1371/journal.pone.0080848

**Published:** 2013-11-18

**Authors:** Hans Dieter Nischalke, Cordula Berger, Philipp Lutz, Bettina Langhans, Franziska Wolter, Marianne Eisenhardt, Benjamin Krämer, Pavlos Kokordelis, Andreas Glässner, Tobias Müller, Jonas Rosendahl, Janett Fischer, Thomas Berg, Frank Grünhage, Ludger Leifeld, Michael Soyka, Jacob Nattermann, Tilman Sauerbruch, Felix Stickel, Ulrich Spengler

**Affiliations:** 1 Department of Internal Medicine I, University of Bonn, Bonn, Germany; 2 Medical Clinic for Hepatology and Gastroenterology, Medical University Charité Campus, Virchow–Klinikum Berlin, Berlin, Germany; 3 Department of Gastroenterology, University Hospital Leipzig, Leipzig, Germany; 4 Department of Medicine II, Saarland University Hospital, Homburg, Germany; 5 Internal Medicine, Evangelisches Krankenhaus Kalk, Cologne, Germany; 6 Psychiatric Hospital Meiringen, Meiringen, Switzerland; 7 Psychiatric Hospital, University of Munich, Munich, Germany; 8 Department of Visceral Surgery and Medicine, Inselspital, University of Bern, Bern, Switzerland; Yonsei University College of Medicine, Korea, Republic Of

## Abstract

**Background and Aims:**

CXCL1 (CXC chemokine-ligand-1) is a ligand for CXC chemokine receptor 2 expressed on hepatic stellate cells (HSC). Thus, CXCL1 might contribute to HSC activation and fibrogenesis. In the present study, we investigated the influence of the *CXCL1* rs4074 polymorphism on the occurrence of alcohol induced liver cirrhosis and hepatocellular carcinoma (HCC).

**Methods:**

The study involved 458 patients with alcoholic cirrhosis (170 with HCC), 115 alcoholics without liver disease and 342 healthy controls. All subjects were genotyped for the *CXCL1* rs4074 polymorphism and CXCL1 serum levels of 132 patients were measured. In vitro CXCL1 secretion in TLR-transfected cell lines were studied by ELISA.

**Results:**

Distribution of the *CXCL1* genotypes (GG/GA/AA) was 159/219/80 in patients with alcoholic cirrhosis, 52/44/19 in alcoholic controls and 158/140/44 in healthy controls. Patients with alcohol-induced cirrhosis were significantly more often carriers of the *CXCL1* rs4074 A allele (65.3%) than alcoholics without liver disease (54.8%, OR=1.55; 95%CI=1.025-2.350; p=0.04) and healthy controls (53.8%, OR=1.62; 95%CI=1.212-2.151; p=0.001). Accordingly, the frequency of the CXCL1 rs4074 A allele was significantly higher in the cirrhotic patients than in the subjects without cirrhosis (41.4% vs. 33.9%, OR=1.38, 95% CI:1.14–1.66, p=0.001). Furthermore cirrhotic carriers of the *CXCL1* rs4074 A allele had significantly higher CXCL1 serum levels than carriers of the GG genotype. In contrast to sera from healthy controls, sera from patients with alcoholic cirrhosis induced CXCL1 secretion in TLR2- (p=0.016) and TLR4- (p=0.008) transfected HEK293 cells. This finding indicates that sera from patients with alcoholic cirrhosis contain soluble ligands that can induce CXCL1 production via stimulation of TLRs.

**Conclusion:**

The enhanced CXCL1 serum levels in carriers of the rs4074 A allele together with their increased frequency in patients with alcohol induced cirrhosis suggest the *CXCL1* rs4074 A allele as a genetic risk factor for alcoholic cirrhosis.

## Introduction

In Western industrialized countries, alcoholic liver disease represents the premier cause of liver cirrhosis. Chronic exposure to harmful amounts of alcohol and its toxic intermediates (acetaldehyde, reactive oxygen species) causes steatosis, necroinflammation and fibrotic remodeling via the release of pro-fibrogenic factors and subsequent functional alterations of innate immune cells (e.g. natural killer cells and Kupffer cells) and hepatic stellate cells (HSC) [1,2].

Numerous intrahepatic chemokines and their receptors are up-regulated in alcohol-induced liver fibrosis [3], and a genetic polymorphism in the *CXCL1* gene (rs4074) has recently been identified as an independent factor predisposing to cirrhosis in patients of European descent with chronic hepatitis C [4]. This risk was mediated by up-regulation of CXCL1 expression in response to TLR2 ligands. In the present study, we wanted to investigate whether this polymorphism predisposes to hepatic fibrotic remodeling also in the context of alcoholic liver disease. CXC chemokine-ligand-1 (CXCL1), previously known as Gro-alpha, is an inflammatory cytokine which binds to the G protein coupled CXCR2 chemokine receptor, promoting fibrogenesis and angiogenesis [5,6,7,8] and thereby exerting pro-oncogenic effects [9,10]. In addition, CXCL1 is considered to play a pivotal role in the stimulation of hepatic stellate cells [2] and acts as a chemoattractant for neutrophils [11]. In alcoholic hepatitis, hepatic expression of CXCL1 is up-regulated and correlates with neutrophil infiltration and the severity of portal hypertension, but in contrast to other cytokines, CXCL1 levels are not associated with a poor clinical outcome [12]. CXCL1 has also been identified as a serum biomarker associated with hepatocellular carcinoma (HCC) [13]. CXCL1 is mainly expressed by mononuclear cells, fibroblasts and several tumor cell lines that release inflammatory chemokines in response to activation of pathogen recognition receptors, e.g. Toll-like receptors (TLR) [14,15,16]. Since increased levels of circulating lipopolysaccharide (LPS) are recognized as pivotal triggers of necroinflammation chronic alcoholic liver disease [17], enhanced production of CXCL1 might be mediated via the LPS receptor TLR4. The CXCL1 gene is located on chromosome 4q12-q13 and features a recently described G/A single nucleotide polymorphism (SNP) at position 74736144 (rs4074). The variant A allele of this SNP was weakly associated with HCC in Japanese patients [18], and showed a strong association with HCV genotype-1 induced cirrhosis in a previous study from our group [4]. The present investigation focused on the potential influence of the CXCL1 rs4074 single nucleotide polymorphism on the occurrence of alcoholic cirrhosis and HCC in a cohort of European descent.

## Materials and Methods

### Ethics Statement

The study conformed to the ethical guidelines of the Helsinki Declaration, as approved by the Institutional Review Boards of the Bonn, Berlin, Bern and Leipzig University Ethics Committees. Written informed consent was obtained from the patients prior to sample collection. Samples were coded and data stored anonymously.

### Study Population and Sample Collection

A total of 288 patients with alcoholic (>300g per week) cirrhosis without HCC (65.7% male; median age 59 years), 170 patients with alcohol-associated cirrhosis with HCC (89.4% male; median age 63 years), 115 alcoholic controls (70.4% male; median age 40.5 years) and 342 healthy controls (51.2% male; median age 58.5 years) participated in this study. Participants with alcoholic cirrhosis were recruited at German University Departments of Hepatology in Bonn, Berlin, Erlangen-Nürnberg and Leipzig between 2005 - 2013. Patients with viral infection (anti HCV positive or anti HBc positive) had been excluded from this study. Cirrhosis was diagnosed by the presence of unequivocal clinical evidence (ascites, hepatic encephalopathy, varices), laboratory results indicating impaired liver function, and imaging findings (computed tomography, ultrasound) compatible with cirrhosis, or transient elastography (cut off 19.5) [19] and, if available, histology. The diagnosis of HCC was made by contrast enhanced magnetic resonance imaging and computed tomography according to established diagnostic criteria [current EASL and AASLD guidelines]. 

Alcoholics without apparent liver disease were selected from a previously reported cohort [20,21] of patients with heavy alcohol abuse (median duration 17 years (10-49); median amount of alcohol consumption 1890g/week (560-6300), who had normal appearance of the liver on ultrasound, normal transaminases and a γglutamyl transpeptidase < 55 U/l. For confirming eligibility of control patients, present alcohol consumption was quantified through interrogation during a face-to-face interview. All patients received a diagnosis of alcohol dependence (per Diagnostic and Statistical Manual of Mental Disorders-IV criteria) by the consensus of two clinical psychiatrists. Furthermore, DNA samples from 342 healthy controls, comprising blood donors (n=144) and healthy participants of colonic cancer-screening programs (n=198) which had indicated to consume less than 300g EtOH per week, were analyzed to check the distribution of genotypes in the background population. All subjects in this study were of European descent, further characteristics are listed in Table 1. 

**Table 1:** Demographic and Clinical Data of the Study Groups.

	**Alcoholic cirrhosis without HCC**	**Alcoholic cirrhosis with HCC**	**Alcoholic controls**	**Healthy controls**
**Total number**	288	170	115	342
**Median age**, years (range)	59 (29-79)	63 (36-87)	40.5 (22-74)*	58.5 (20-86)*
**Gender** (male/female)	65.7% / 34.3%	89.4% / 10.6%	70.4% / 29.6%	51.2% / 48.8%*
**Mean** BMI ± SD	26.0 ± 5.9	27.2 ± 4.7	24.0 ± 3.9*	-
**Mean** MELD ± SD	16.5 ± 7.6	17.5 ± 9.0	-	-
**Mean γ-**GT ± SD	199.3 ± 223.0	275.2 ± 293.4	30.2 ± 13.6*	-
**Mean** AST ± SD	84.3 ± 262.6	105.8 ± 136.5	23.7 ± 17.4*	-
**Mean** ALT ± SD	47.5 ± 166.4	50.4 ± 57.4	21.9 ± 18.6*	-
**Mean Bilirubin** ± SD	4.32 ± 7.07	3.36 ± 4.67	0.62 ± 0.38*	-
**Platelet count** [*10^3^/µl], (Mean ± SD)	153.7 ± 96.2	147.9 ± 89.0	260.0 ± 74.3*	-

* p<0.05 vs. alcoholic cirrhosis without HCC and vs. Alcoholic cirrhosis with HCC

### Determination of rs4074 alleles

Genomic DNA was extracted from 200 μL EDTA-blood using the QIAamp Blood Mini Kit (Qiagen, Hilden, Germany) according to the manufacturer’s protocol. 

Determination of the *CXCL1* rs4074 polymorphism was performed by LightCycler real-time PCR using a LightSNiP (SimpleProbe) assay from TIB-MolBiol (Berlin, Germany). Samples were set up in a final volume of 10 µl, containing 1 µl of DNA solution, 2 µl of LightCycler FastStart DNA MasterPLUS HybProbe Mix (Roche Molecular Biochemicals, Mannheim, Germany), and 1µl of LightSNiP reagent mix (Tib MolBiol, Berlin, Germany). The cycling conditions were as follows: initial denaturation at 95°C for 10 min, followed by 45 cycles of denaturation at 95°C for 10 sec, annealing at 60°C for 10 sec, and extension at 72°C for 15 sec. Fluorescence was monitored at the end of each annealing phase at 60°C. After completion of the PCR, a melting curve of the amplification products was plotted by denaturation at 95°C for 20 sec, holding the sample at 40°C for 20 sec, and then slowly heating the sample to 85°C with a ramp rate of 0.2°C/sec and continuous fluorescence acquisition.

### Determination of CXCL1 serum levels

CXCL1 concentrations in patient sera and in cell culture supernatants were assessed by the human CXCL1/GROalpha Quantikine ELISA (R&D Systems, Wiesbaden, Germany).

### Stimulation of TLR-transfected HEK293 cells with alcoholic and healthy sera

To investigate whether CXCL1 induction in patients with alcoholic cirrhosis is triggered via TLR´s, 200,000 TLR2- or TLR4-transfected and un-transfected HEK293 cells were incubated over 24h with sera (50% v/v) from healthy controls and patients with alcoholic cirrhosis on 48-well plates (500 µL/well) in a humidified atmosphere (37°C with 5% CO_2_). TLR-transfected and control HEK293 cells were kindly provided by G. Szabo, University of Massachusetts, Worcester, MA. CXCL1 concentrations in the supernatants were determined by ELISA, and measurements were done in duplicates. 

### Stimulation of primary HSCs with recombinant CXCL1

Stimulation experiments were performed to check if CXCL1 can induce pro-fibrotic genes such as collagen I and alpha smooth muscle actin (α-SMA). Isolated human primary HSCs (pHSC, ScienCell, Carlsbad, CA, USA) were used between passage 2 and 6, and cultured in defined medium supplemented with 2% fetal bovine serum and 50  U/ml penicillin and 50  μg/ml streptomycin (all ingredients obtained from ScienCell). 50,000 pHSCs were incubated over 16h with 250 pg/ml recombinant CXCL1 (BioLegend, San Diego, CA, USA) on 48-well plates (500 µL/well) in a humidified atmosphere (37°C with 5% CO_2_). After 2 hours 5µg/ml brefeldin A (BD Bioscience, Germany) were added to block secretion of collagen and αSMA. After CXCL1 stimulation, cells were permeabilized (BD Cytofix/Cytoperm™, BD Bioscience) and intracellularly stained with Anti-Collagen Type I-FITC Antibody (clone 5D8-G9; Merck KGaA, Darmstadt, Germany) or goat anti-human α-SMA (ABIN185271; antibodies-online GmbH, Aachen, Germany) with secondary anti-goat-NL577-conjugated antibody (R&D Systems GmbH; Wiesbaden-Nordenstadt, Germany) on a BD FACSCanto flowcytometer using the CellQuest Pro (BD Biosciences) and FlowJo 7.2.2 software packages (TreeStar Inc., Ashland, OR). Expression was analyzed in comparison to un-stimulated HSC.

### Statistical analysis

Differences between groups were analyzed by t-test and Mann-Whitney-U test as appropriate. 

Statistical analysis was performed with SPSS 17.0 (SPSS, Munich, Germany). Data are given as means ± SD, unless stated otherwise.

Genotype frequencies were determined and tested for consistency with Hardy-Weinberg equilibrium using an exact test. Allele and genotype frequencies were compared between cases and controls by Pearson's goodness-of-fit Chi^2^ test and Armitage's trend test, respectively (http://ihg.gsf.de/cgi-bin/hw/hwa1.pl). For the statistical comparison of carriers and non-carriers we used Fisher’s exact test. Sample size calculation was done using Lenth, R. V. (2006-9) Java Applets for Power and Sample Size [Computer software], retrieved from http://www.stat.uiowa.edu/~rlenth/Power. We hypothetically extrapolated data from our previous publication [4] concerning *CXCL1* rs4074 variants in HCV-related liver disease. With 342 controls, a sample size of 452 cases was calculated to ensure 80% statistical power at 5% alpha error.

To take into account further potential confounding risk factors of liver cirrhosis the effects of age, gender and *PNPLA3* genotype were assessed by univariate comparisons (ANOVA and chi^2^-statistics) followed by multivariate logistic regression with forward conditional variable selection. Parameters with effects at p<0.1 were entered into the multivariate analysis.

## Results

### Study population

**Figure 1:**
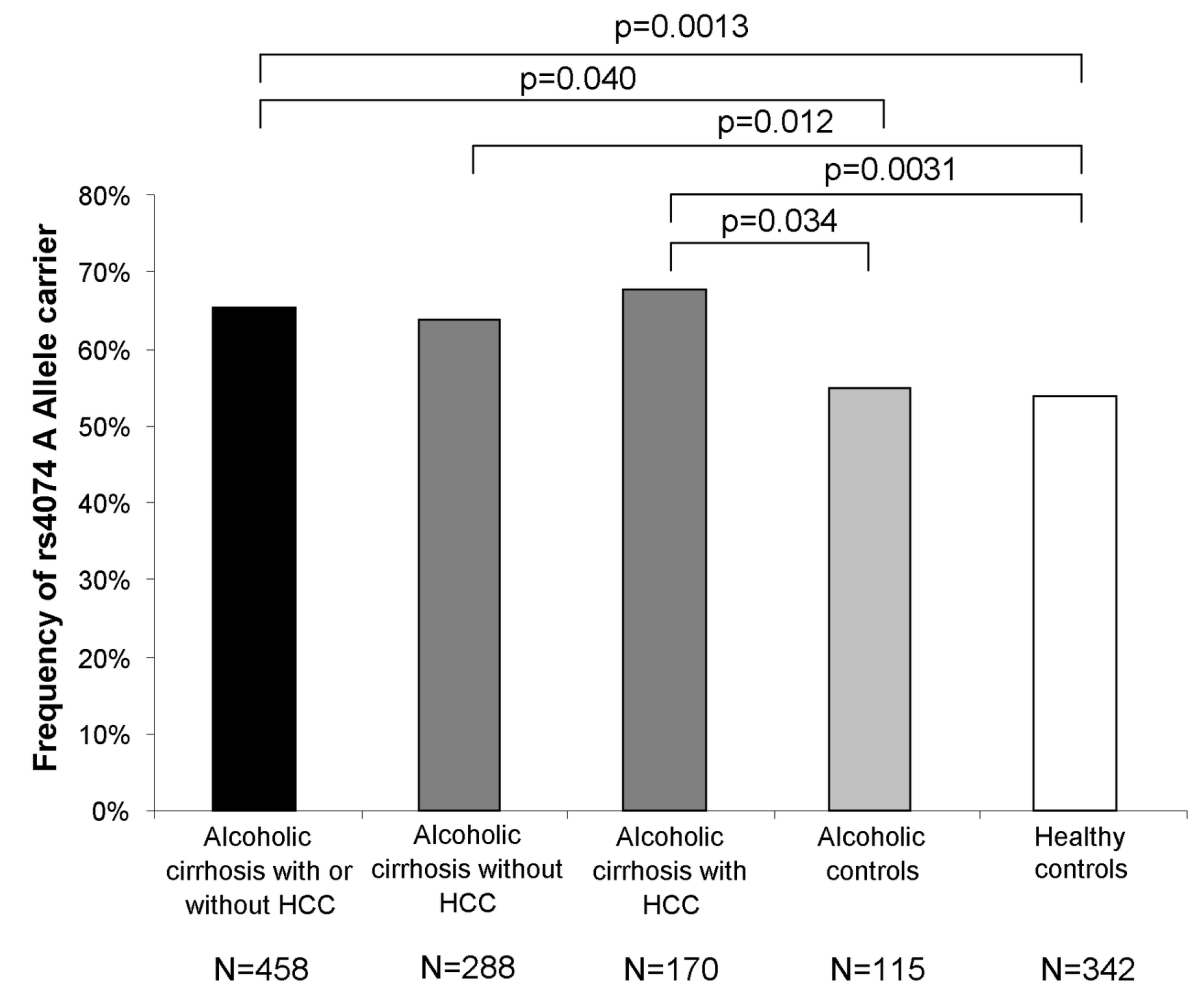
Frequency of the rs4074 A allele in the study groups. This figure illustrates that carriers of the *CXCL1* *rs4074* A allele were equally frequent in patients with alcohol related cirrhosis without and with HCC, but overrepresented in comparison to patients with alcohol abuse without liver damage and to healthy controls. Statistical significances refer to Fisher´s exact test.

The *CXCL1* rs4074 gene polymorphism was determined in all 915 participants and genotype frequencies were consistent with the Hardy–Weinberg equilibrium in all study groups. Distribution of *CXCL1* genotypes (GG, GA, AA) was 159 (34.7%), 219 (47.8%), 80 (17.5%) in cirrhotic patients with or without HCC, 52 (45.2%), 44 (38.3%), 19 (16.5%) in alcoholic controls and 158 (46.2%), 140 (40.9%), 44 (12.9%) in healthy controls. Thus carriage of the *CXCL1* rs4047 A allele was observed significantly more frequently among patients with alcoholic cirrhosis (65.3%) than in alcoholic controls (54.8%, OR=1.55; 95%CI=1.025-2.350; p=0.04) or healthy controls (53.8%, OR=1.62; 95%CI=1.212-2.151; p=0.001). (Figure 1). The frequency of carriers of the A allele was comparable in cirrhotic patients with or without HCC (63.9% vs. 67.6%; p=0.48). Furthermore severity of liver cirrhosis, indicated by the model of end-stage liver disease (MELD) score, was comparable between patients who carried the A allele of the *CXCL1* rs4047 polymorphism and patients without this allele (mean 16.7 versus 16.8; p=0.639).

To check if carriage of the *CXCL1* rs4047 A allele is an independent risk factor for alcoholic cirrhosis when other known risk factors such as age, gender, and the *PNPLA3* rs738409 risk variant were also taken into account, we calculated a Cox regression model (table 2). This analysis confirmed carriage of the *CXCL1* rs4047 A allele as an independent risk factor for alcoholic cirrhosis (OR 1.485; 95%-CI: 1.084-2.035; p=0.014). Furthermore, age, gender and *PNPLA3* risk variant were confirmed as risk factors for alcoholic cirrhosis.

**Table 2:** Regression Analysis for possible risk factors contributing to alcoholic cirrhosis.

Univariate Analysis				
			**95% CI**	
**Parameter**	**P**	**OR**	**Lower**	**Upper**
Age (per year)	<0.00001	1.050	1.039	1.061
CXCL1 risk variant (GA/AA)	0.00059	1.599	1.225	2.086
Gender (male)	<0.00001	2.543	1.898	3.406
PNPLA3 risk variant (IM/MM)	<0.00001	2.814	2.152	3.679
			**95% CI**	
**Parameter**	**P**	**OR**	**Lower**	**Upper**
Age (per year)	<0.00001	1.050	1.038	1.062
CXCL1 risk variant (GA/AA)	0.014	1.485	1.084	2.035
Gender (male)	<0.00001	2.389	1.717	3.326
PNPLA3 risk variant (IM/MM)	<0.00001	2.657	1.946	3.630

Multivariate Analysis*

* (including all significant parameters from the univariate analysis)

CI, confidence interval; OR, odds ratio;

### CXCL1 rs4074 gene polymorphism and serum CXCL1 levels

**Figure 2:**
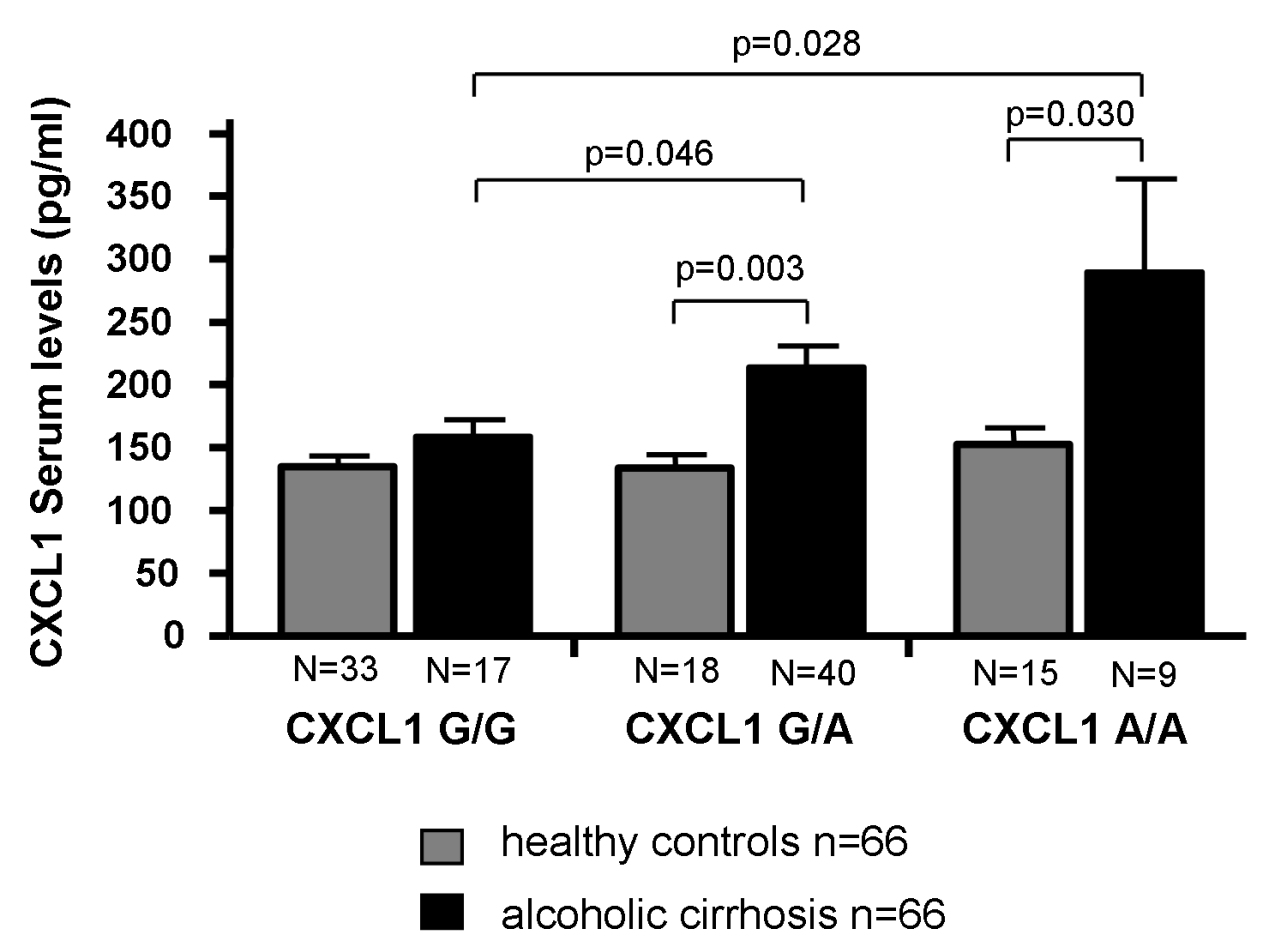
CXCL1 serum levels in patients with alcoholic cirrhosis and healthy controls with distinct *CXCL1* **rs4074 genotypes**. This figure shows CXCL1 serum concentrations in 66 healthy controls (grey columns) and in 66 patients with alcoholic cirrhosis (black columns) stratified with respect to rs4074 variants G/G, G/A and A/A. Results are shown as means ± standard errors. Groups were compared with the Mann-Whitney U test.

To determine whether the *CXCL1* rs4074 G/A polymorphism influences the CXCL1 expression in patients with high alcohol consumption (>300g per week), we measured CXCL1 serum levels in 66 samples of patients with alcoholic cirrhosis and healthy controls, respectively (Figure 2). In healthy controls, CXCL1 serum levels did not differ between carriers of the diverse rs4074 genotypes (GG = 134.9 ± 8.0 pg/ml, GA = 133.7 ± 10.5 pg/ ml, AA = 152.4 ± 13.4 pg/ml). In alcoholic patients with the homozygous rs4074 G/G genotype, CXCL1 serum levels corresponded to the levels detected in the healthy controls (157.9 ± 13.2 pg/ml). However, patients with alcohol-induced liver cirrhosis carrying the rs4074 A allele had significantly higher CXCL1 serum levels (GA = 212.7 ± 16.5 pg/ml (p = 0.034); AA = 287.9 ± 73.3 pg/ml (p=0.028) than patients with the GG genotype and in comparison to the corresponding genotypes of the healthy controls (GA = 133.7 ± 10.5 pg/ml, p=0.003; AA = 152.4 ± 13.4 pg/ml, p=0.03) 

### Stimulation of TLR2- and TLR4-transfected HEK293 cells with sera from patients with alcoholic liver cirrhosis

**Figure 3:**
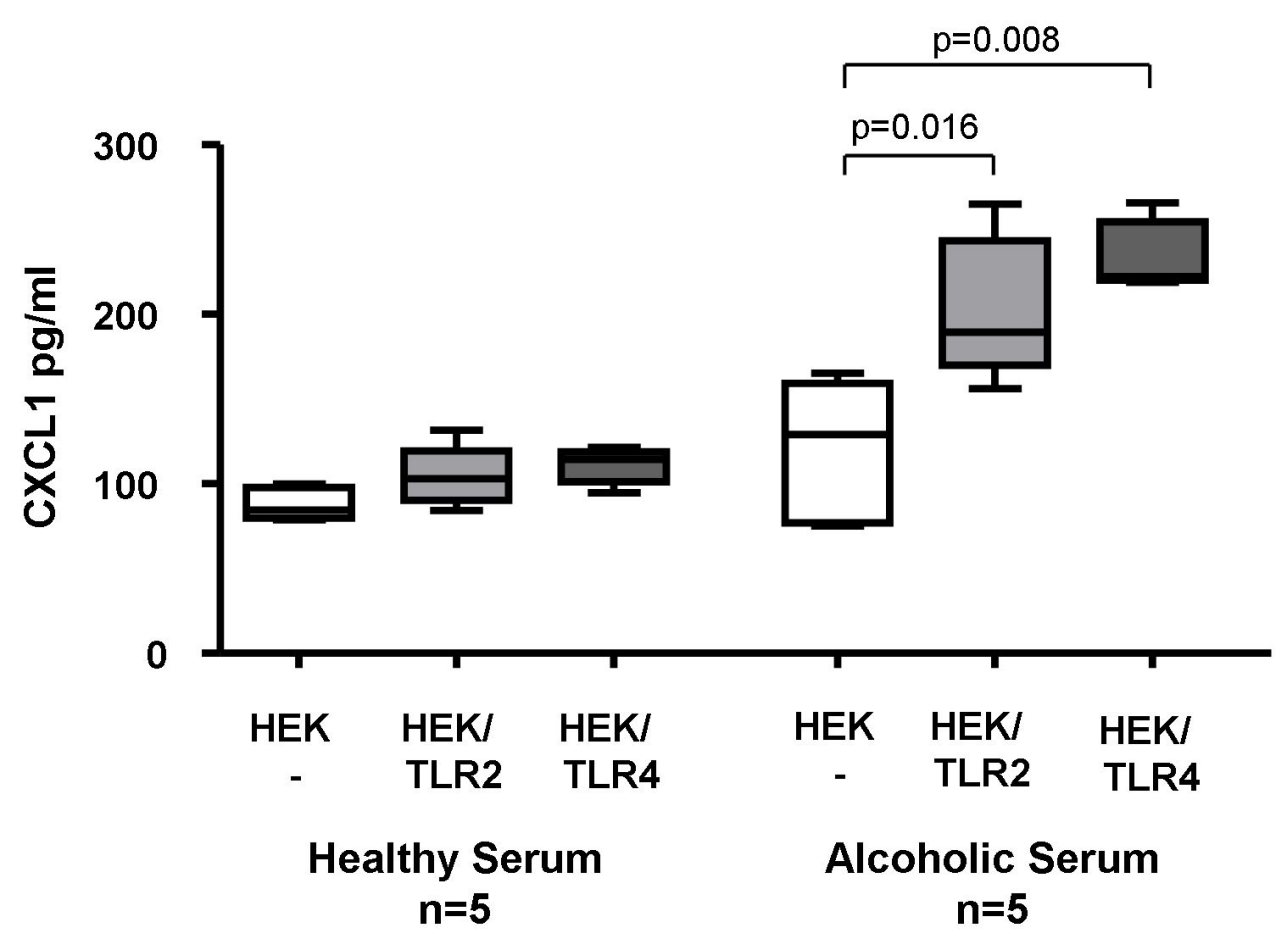
In Vitro CXCL1 induction in TLR2- and TLR4-transfected HEK293 cells. HEK293 cells stably transfected with human TLR2 (HEK/TLR2) and human TLR4 (HEK/TLR4) as well as untransfected HEK293 cells (HEK -) were incubated with sera from patients with alcoholic cirrhosis (box plots on the right side) and sera from healthy controls (box plots on the left side). Stimulation with sera from cirrhotic patients showed significantly enhanced CXCL1 secretion in the TLR2- and TLR4-transfected cells but not in the un-transfected control cells. In contrast, sera from healthy controls did not up-regulate CXCL1 secretion in the TLR2- and TLR4-transfected HEK293 cells. P values were calculated using Mann-Whitney U test.

Diverse ligands such as LPS and peptidoglycan in serum of alcoholic patients are known to activate TLR2 and TLR4 [22]. To assess whether stimulation of these pattern recognition receptors contribute to CXCL1 induction in alcoholics, we stimulated TLR2- and TLR4-transfected HEK293 cells as well as untransfected control cells with sera from heavy drinkers and healthy controls, respectively. Whereas sera from healthy controls could not induce significant changes in CXCL1 levels in the supernatants of TLR-transfected HEK293 cells, sera from alcoholics resulted in increased CXCL1 expression both in TLR2-transfected (p=0.016) and TLR4-transfected cells (p=0.008) but not in the un-transfected HEK293 cell line (Figure 3). Thus, sera of alcoholic patients contained ligands stimulating CXCL1 secretion both via TLR2 and TLR4 signaling pathways. 

### Induction of pro-fibrotic genes by CXCL1

**Figure 4:**
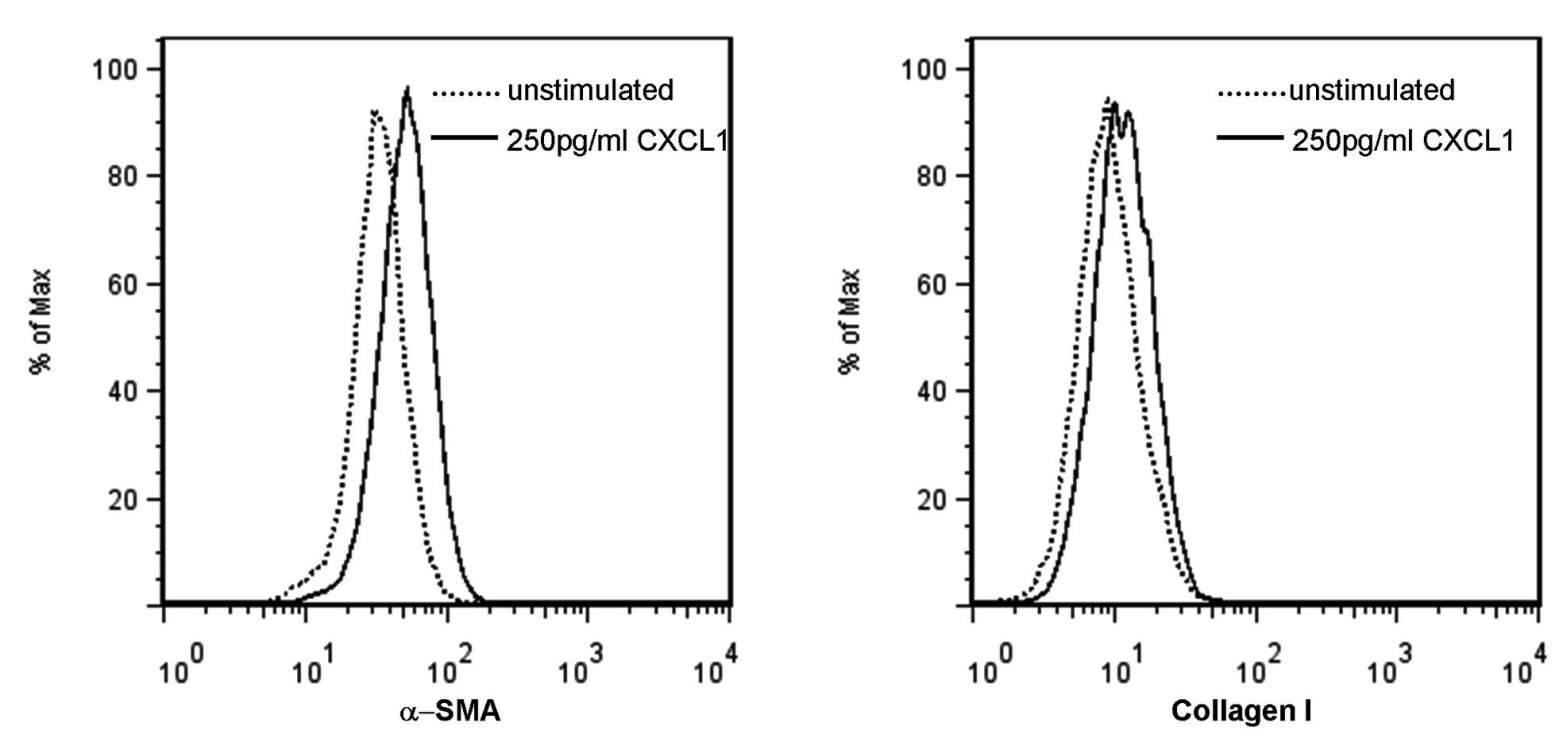
Upregulation of fibrosis markers in human HSC after stimulation with CXCL1. After incubation with and without 250pg/ml recombinant CXCL1 for 16 hours, human HSC were stained for α-SMA and Collagen type I and analysed by flow cytometry. This representative set of histograms shows that α-SMA and Collagen type I expression increases after CXCL1 stimulation (solid lines) as compared to the unstimlated controls (dotted line).

In order to confirm a putative upregulation of surrogate markers of liver fibrogenesis from increased CXCL1 levels in vitro, hepatic stellate cells were incubated with 250pg/ml recombinant CXCL1. The production of α-SMA and collagen I was clearly increased in comparison to non-stimulated HSC’s (Figure 4). 

## Discussion

Alcohol is a major cause of chronic liver disease worldwide and comprises various grades of liver damage, ranging from steatosis and steatohepatitis, either with or without fibrosis, to fully established cirrhosis and eventually HCC. The present study addressed the question, whether the *CXCL1* rs4074 polymorphism predisposes to liver cirrhosis in patients with chronic alcohol abuse (>300g/week). Our cross-sectional study revealed that the CXCL1 A allele is more frequent among patients with alcoholic cirrhosis compared to healthy controls and to patients with alcohol abuse in the absence of liver damage. Multivariate analysis confirmed the *CXCL1* rs4074 risk variant as independent risk factor for alcoholic liver cirrhosis in addition to age, gender and the *PNPLA3* 148M risk variant. Unlike the PNPLA3 148M variant which increases the risk for cirrhosis as well as HCC [23], the CXCL1 rs4074 polymorphism did apparently not infer an additional risk for HCC, since frequencies of the CXCL1 A allele in the alcoholic cirrhotics with and without HCC did not differ significantly. In contrast, a Chinese research group identified CXCL1 together with thrombin light chain and alpha-fetoprotein as serological biomarkers for HCC in hepatitis B infected patients [13]. This discrepancy could possibly be explained by the different ethnic background, and differences in the pathogenesis between HBV and alcohol induced cirrhosis. Pathogenesis of cirrhosis in the context of chronic alcohol abuse reflects a complex interplay between immune cells, inflammatory cytokines, hepatocytes, and mesenchymal cells. Nevertheless, activation of HSC seems to represent a final, general step and results in the secretion of excess extracellular matrix molecules. CXCL1 is a chemokine preferentially expressed by monocytes. It binds to the CXC chemokine receptor 2 (CXCR2) expressed on HSC and neutrophils [24,25]. In liver biopsies of patients with alcoholic hepatitis, a Spanish research group recently described up-regulation of CXCL1 expression in correlation with neutrophilic infiltration and the severity of portal hypertension [12]. Apart from this indirect cytopathic effect via attraction of neutrophils, stimulation of murine HSC with recombinant KC, a mouse homologue of human CXCL1, resulted in increased collagen type I production, up-regulation of CXCR2 and pronounced cell death of mouse hepatocytes. This indicates a supplementary direct hepatotoxic effect of CXCL1 and its receptor CXCR2 with a positive feedback mechanism [24,26]. Similarly, our stimulation experiments show that CXCL1 upregulates collagen type I and α-SMA in human HSC’s. Beyond that, other CXCR2 ligands such as interleukin (IL)-8 are known to strongly stimulate fibrogenesis [27]. Thus, genetic variants in the IL8 (CXCL8) gene have also been proposed to modulate the risk of pulmonary fibrosis in cystic fibrosis [28]. 

The present study suggests that the rs4074 single nucleotide polymorphism within the *CXCL1* gene is associated with increased CXCL1 blood levels and increased risk for development of cirrhosis in alcoholics. Besides the statistical association, our analysis provides mechanistic findings that yielded a potential way by which the *CXCL1* rs4074 polymorphism may contribute to fibrotic remodeling. Sera from alcoholic patients, but not from healthy controls, were able to stimulate TLR2- and TLR4-transfected HEK293 cells to secrete CXCL1, suggesting that CXCL1 up-regulation is dependent on soluble factors found in alcoholic liver disease, and involves TLR2 and TLR4 signaling. A potential TLR4 ligand in the serum of alcoholic patients could be LPS, which can be found both in blood of alcoholic patients as well as in rodents exposed to ethanol [17]. LPS can promote fibrogenesis indirectly via CD14/TLR4 on Kupffer cells with NFkappaB activation and secretion of profibrogenic factors [29,30] and directly via TLR4 on HSC [31,32]. Potential TLR2 agonists in alcoholic patients are presumably the bacterial peptides peptidoglycan and zymosan. Alcohol leads to leaks in the intestinal barrier, so that concentrations of TLR2 agonists increase in the portal blood with subsequent spill-over to the systemic circulation [22]. According to Oak and colleagues, augmentation of inflammatory response depends on the simultaneous presence of TLR2 and TLR4 ligands and involves IRAK1-activation and phosphorylation of Janus kinase [33]. 

The molecular mechanism responsible for the different levels of CXCL1 expression as a function of the *CXCL1* rs4074 polymorphism remains unknown. However, we recently discussed an additional transcription factor binding site for interferon-regulatory factor-3 (IRF-3) and signal transducer and activator of transcription (STAT) 5a [4]. 

Although our study is limited by its cross-sectional design it nevertheless provides first experimental evidence suggesting the *CXCL1* rs4074 polymorphism modulates the risk for cirrhosis in Caucasian patients with high alcohol consumption. Thus it provides the rationale to warrant a prospective controlled long-term study to define this risk more precisely. In addition, our findings may contribute to a deeper understanding of liver fibrosis and the development of antifibrotic drugs.
